# Embryo Aggregation in Pig Improves Cloning Efficiency and Embryo Quality

**DOI:** 10.1371/journal.pone.0146390

**Published:** 2016-02-19

**Authors:** Carla Paola Buemo, Andrés Gambini, Lucia Natalia Moro, María Inés Hiriart, Rafael Fernández-Martín, Philippe Collas, Daniel Felipe Salamone

**Affiliations:** 1 Laboratory of Animal Biotechnology, Faculty of Agriculture, University of Buenos Aires, Buenos Aires, Argentina; 2 National Institute of Scientific and Technological Research, Buenos Aires, Argentina; 3 Department of Molecular Medicine, Institute of Basic Medical Sciences, Faculty of Medicine, University of Oslo, and Norwegian Center for Stem Cell Research, Oslo, Norway; Michigan State University, UNITED STATES

## Abstract

In this study, we analyzed the effects of the cloned embryo aggregation on *in vitro* embryo development and embryo quality by measuring blastocyst diameter and cell number, DNA fragmentation levels and the expression of genes associated with pluripotency, apoptosis, trophoblast and DNA methylation in the porcine. Zona-free reconstructed cloned embryos were cultured in the well of the well system, placing one (1x non aggregated group) or three (3x group) embryos per microwell. Our results showed that aggregation of three embryos increased blastocyst formation rate and blastocyst diameter of cloned pig embryos. DNA fragmentation levels in 3x aggregated cloned blastocysts were significantly decreased compared to 1x blastocysts. Levels of *Oct4*, *Klf4*, *Igf2*, *Bax* and *Dnmt 1* transcripts were significantly higher in aggregated embryos, whereas *Nanog* levels were not affected. Transcripts of *Cdx2* and *Bcl-xl* were essentially non-detectable. Our study suggests that embryo aggregation in the porcine may be beneficial for cloned embryo development and embryo quality, through a reduction in apoptotic levels and an improvement in cell reprogramming.

## Introduction

Mammalian cloning by somatic cell nuclear transfer has an enormous potential in biotechnology and has opened new possibilities for specific genetic modifications in farm animals [1]. In addition to the potential application in agriculture and biomedicine, somatic cell nuclear transfer (SCNT) is one of the most powerful tools used to study events that occur during reprogramming and cellular differentiation [2,3]. Successful cloning using mammalian somatic cell indicates that epigenetic modifications in the differentiated nucleus can be remodeled to a totipotent state [2]. The first living cloned piglet obtained by nuclear transfer of adult granulosa cells was reported in 2000 [4], and subsequently, additional porcine clones have been obtained [5–7].

Recently, our laboratory has demonstrated the advantage of zona free cloned embryo aggregation in the equine and the feline [8–10]. Embryo aggregation consists of placing more than one zona free embryos in the same well; some of the reported benefits in mammals are: improvement in embryo development in pig [6], equine [8, 9], feline [10] and mouse [11], increasing the density of cells in the inner cell mass on cattle [12], blastocyst diameter in equine and in bovine [8, 13], reduction in apoptosis in bovine and pigs [13, 14, 15], normalization of gene expression in bovine [15]and in some cases, it has also improved *in vivo* embryo development and early pregnancy rates in mouse [11], equine [8] and in cattle [13, 16, 17]. As a single clone may have epigenetic defects [15], embryo aggregation could compensate the reprogramming deficiencies through the interactions between blastomeres [11] or by paracrine pathways [11]. Indeed, it has been proposed that such interaction could also improve post implantation development [18].

During normal embryogenesis a type of programmed cell death called apoptosis occurs as a physiological process and it has a role in the cellular response to suboptimal developmental conditions and stress [19]. Apoptosis helps in the removal of cells with chromosomal abnormalities or inappropriate developmental potentials [20]. However, embryo development could be compromised if apoptosis surpasses a certain threshold [19, 21, 22]. Moreover, its occurrence in preimplantation embryos has been considered one of the most important parameters for evaluation of embryo health and regulated embryo cell numbers [23–25]; the TUNEL assay (terminal deoxynucleotidyltransferase-mediated dUTP nick-end labelling) has been used for detection of apoptosis process in mammalian embryos [20].

Epigenetic mechanisms have shown to critically influence embryonic development through the control of gene expression and chromatin packaging [26] and several genes are reported to be associated with the pluripotency of the embryo: *Oct4* [27], *Sox2* [28] and *Nanog* [29] and with lineage segregation like *Cdx2* [30]. However, porcine embryos present numerous differences in their expression profile of pluripotency markers compared to human or mouse. This may suggest that different mechanisms are implicated in the regulation of pluripotency in this species [31].

To improve the efficiency of porcine cloning, it is necessary to produce high-quality cloned blastocyst [12, 32, 33]. We hypothesized that embryo aggregation could improve the developmental competence and quality of cloned pig embryos. To this aim, we studied the embryo development rates, the total number of cells per blastocyst, the embryo diameter, the DNA fragmentation levels by TUNEL assay and the expression of genes associated with pluripotency, apoptosis, trophoblast and methylation in aggregated and non-aggregated cloned embryos.

## Materials and Methods

### Chemicals

Except otherwise indicated, all chemicals were obtained from Sigma Chemicals Company (St. Louis, MO, USA).

### Recovery and *in vitro* maturation of oocytes

Ovaries were collected from gilts at a local slaughterhouse called "La Pompeya" located in the province of Buenos Aires and transported to the laboratory at around 25–30°C within 3h of collection. Cumulus-oocyte complexes (COCs) from follicles 3 to 6mm in diameter were aspirated using an 18-gauge needle attached to a 10mL disposable syringe. Compact COCs were selected and matured in 100μL drops of tissue culture medium (TCM-199- 31100–035; Gibco, Grand Island, NY, USA) under mineral oil (M8410), supplemented with 0.3mM sodium pyruvate (P2256), 100μM cysteamine (M9768), 5μg/ml myoinositol, 1μg/ml insulin transferrin selenium (ITS; product no. 51300–044; Gibco), 1% antibiotic-antimicotic (15240–096 Gibco), 10% (v/v) porcine follicular fluid, 5 ng/ml basic fibroblast growth factor (bFGF) and 10μg/ml of follicle-stimulating hormone (FSH) (NIH-FSH-P1Folltropin, Bioniche, Caufield Junction Caufield North, Victoria, Australia). Maturation was performed at 39°C in a humidified atmosphere of 6.5% CO2 in 90% air for 42-44h.

#### Preparation of oocytes for SCNT

The cumulus cells were removed by vortexing COCs for 3–4 min in hyaluronidase (H-4272) solution: 1 mg/ml in Tyrode’s Lactate-Pyruvate-HEPES medium (TALP-H). Nuclear maturation was confirmed by the presence of the first polar body. Matured oocytes were incubated in 1.5 mg/ml pronase (P-8811) in TALP-H for 1–2 min to remove the zona pellucida (ZP). Zona Free (ZF)-oocytes were kept in SOF until enucleation.

#### Somatic cell nuclear transfer

ZF- oocytes were incubated for 15 minutes in a SOF drop containing 1μg/mL Bisbenzimide H 33342 (B2261) and 0.5μg/ml of cytochalasin B. The metaphase phase was aspirated using a blunt pipette under UV light, and a closed holding pipette was used to support the oocyte during the enucleation. Successful enucleation was assessed by the observation, under UV light, of the entire metaphase plate inside the pipette [8]. ZF-enucleated oocytes were kept in SOF medium under culture conditions until nuclear transfer.

Landrace fetal fibroblast cells was established from a primary culture from fetal lungs of approximately 30 days and left to culture until 80% of confluence. Afterwards, cells were frozen in Dubelcco′s modified Eagle medium (DMEM) (11885, Gibco, Grand Island, NY, USA) containing 10% dimethyl sulfoxide. Three days before SCNT, cells were thawed, cultured until confluence and subsequently used between passages 1–4. Single cell suspension was prepared by trypsinization of the cultured cells and then resuspended in DMEM medium supplemented with 20% fetal bovine serum (FBS; 10499–044, Gibco) and 1% antibiotic–antimycotic (15240–096, Gibco) and stored in liquid nitrogen.

ZF-enucleated oocytes were individually transferred to a 40μl drop of 1mg/ml phytohemagglutinin (L8754) dissolved in TCM-199. After a few seconds, oocytes were quickly dropped over a single donor cell resting on the bottom of a 100 μl TALP-H drop. Afterward, the couplets were placed in fusion medium [0.3 M mannitol (M9546), 0.1 mM MgSO4 (M7506), 0.05 mM CaCl^2^ (C7902), and 1 mg/ml polyvinyl alcohol (P8136)] for 2–3 min and then transferred to a fusion chamber containing 2 ml of fusion medium. Electrofusion was performed using a BTX Electro-Cell Manipulator 830 (BTX, Inc., San Diego, CA) with a double-direct current pulse of 1.42 kV/cm, in which each pulse lasted for 30 μsec. Immediately after, couplets were individually placed in 5 μl microdrop of SOF medium and incubated under mineral oil at 39°C in 5% CO2 in air. Twenty to thirty minutes after the pulse, each couplet was considered fused when the donor cell was not observed in the droplet. Non-fused couplets were re-fused. Non-fused couplets after the second round of fusion were discarded.

Two hours after fusion, ZF-fused couplets were electrically activated by a single direct current pulse of 1.2 kV/cm for 80 μsec, followed by incubation for 3h of 2 mM 6-dimethylaminopurine (D2629) in a 100μl drop of SOF medium.

### *In vitro* embryo culture and embryo aggregation

ZF-reconstructed embryos (ZFREs) were cultured in SOF medium in a humidified atmosphere of 5% CO2 and 95% air at 38.5°C for 7 days. A slightly modified well of the well system [34] was used to culture ZFREs [8]. Briefly, microwells were produced using a heated glass capillary slightly pressed to the bottom of a 35 mm x 10mm petri dish. Microwells were covered with a 50μl microdrop of SOF medium. Embryo aggregation was performed by placing randomly more than one ZFRE per microwell. Two different experimental groups were performed: group 1x, one ZFRE per microwell (non-aggregated embryos; control group); and group 3x, three ZFREs per microwell. The total number of ZFREs per microdrop was similar between groups. Embryos were cultured in a humidified gas mixture (5% CO2, 5%O2, 90% N2) at 38.5°C. Half of the medium was renewed at day 2 with fresh SOF, and at day 5 the medium was renewed again with SOF medium containing 10% FBS. Cleavage was assessed 48 h after activation, and blastocyst formation and blastocyst diameter were recorded on day 7 when the embryos were either fixed for TUNEL assay or stored in RNAlater (AM7020, Ambion Co., Austin, TX, USA) for gene expression study.

### DNA fragmentation levels in blastocysts: TUNEL assay

Pig cloned blastocyst from both experimental groups were fixed in 4% paraformaldehyde (F1635) and then washed and stored in Dulbecco′s phosphate buffered saline (DPBS) (14287–072 Gibco, Gran Island, NY) solution with 1 mg/ml of bovine serum albumin (BSA). DNA fragmentation levels were detected in situ using the Dead End TM Fluorometric TUNEL System (terminal deoxynucleotidyltransferase (TdT)-mediated dUTP nick end labelling) (Promega G3250, Madison, WI, USA). Fixed embryos were permeabilized with 0.2% Triton X-100 in DPBS for 15 min at room temperature and rinsed in 0.4% DPBS-BSA. Then, samples were placed in incubation buffer consisting of equilibration buffer, a nucleotide mix containing fluorescein-dUTP and terminal deoxynucleotidyltransferase for 2 h at 39°C in dark. For negative controls, the terminal deoxynucleotidyltransferase was omitted from the reaction. For nuclei counterstain embryos were incubated with 0.5% propidium iodide for 10 min at room temperature, and then washed in BSA solution and finally mounted on a glass slide in 70% v/v glycerol under a coverslip. Blastocysts were analyzed on a Nikon Confocal C.1 scanning laser microscope. An excitation wavelength of 488 nm was selected for detection of fluorescein-12-dUTP and a 544 nm wavelength to excite propidium iodide. Images of serial optical sections were recorded every 1.5–2 μm vertical step along the Z-axis of each embryo. Three-dimensional images were constructed using software EZ-C1 3.9. Total cell numbers and DNA-fragmented nuclei were counted manually. The results were expressed as quantity from the relation between the number of cells with fragmentation of DNA and the number of total cells. The apoptotic index (IAp) was determined, using the following formula: IAp = TUNEL (+) cells * 100/ Total cell number. The apoptotic index in each blastocyst was calculated as the ratio of TUNEL-positive nuclei to total nuclei in each blastocyst and then an unpaired T-Student test was used to calculate the statistic differences between the index of different groups (p<0.05).

### Real-time reverse transcription polymerase chain reaction

For gene expression analysis we used two groups (clone 1x and aggregated 3x clones). We pooled n = 12 blastocyst of each group and repeat this process three times because we did three biological replicates, each repetitions by triplicate. Embryos were washed twice in DPBS to eliminate the RNAlater in which were conserved. RNA was isolated using the RNeasy micro kit (Qiagen, Hilden, Germany, Cat. No.74004) according to the manufacturer’s instruction. All the samples were treated with DNase I (0.04 U/μl) for genomic DNA digestion. After that, RT PCR was performed in 20μl of final volume. Quantitative PCR was applied using SYBR according to the manufacturer’s instructions with MyiQ Single Color Real Time PCR detection System by BIO RAD cycler. Quantification of all gene transcripts was performed by real-time reverse transcription polymerase chain reaction (PCR) using *ACTB* as an internal standard. The reaction mixture (total 12.5 μL) contained 6.5 μl master mix, 0.25 μl of each primer (20 mmol/ul), 5 μL cDNA template (250 ng final concentration), and 0.5 μl Milliq water.

### Primer design

Primers used for expression analysis were designed using Primer3 online version based on available sequences from the database of GenBank (NCBI). Primers and products sizes are shown in Table 1.

**Table 1:** Primers sequences and conditions for RT-qPCR.

Genes	Primer sequences (5–3)	Size of PCR products (bp)	Tm (°C)	Reference or sequence accession numbers
*Klf4*	CCATGGGCCAAACTACCCAC	81	60	NM_001031782.2
*Klf4*	GGCATGAGCTCTTGGTAATGG	81	59	NM_001031782.2
*Nanog*	CCACTGGCCAAGGAATAGCA	88	60	NM_001129971.1
*Nanog*	CAGGCATCCTTGGTGGTAGG	88	60	NM_001129971.1
*Oct4*	GCTCACTTTGGGGGTTCTCT	80	59	NM_001113060
*Oct4*	TGAAACTGAGCTGCAAAGCC	80	59	NM_001113060
*Bcl-xl*	GTTGACTTTCTCTCCTACAAGC	277	62	SUN HWANG, 2008
*Bcl-xl*	GGTACCTCAGTTCAAACTCATC	277	62	SUN HWANG, 2008
*Bax- α*	ACTGGACAGTAACATGGAGC	294	63	SUN HWANG, 2008
*Bax- α*	GTCCCAAAGTAGGAGAGGAG	294	63	SUN HWANG, 2008
*Dnmt1*	TTCTCACTGCCTGACGATGT	79	59	NM_001032355.1
*Dnmt1*	CCTTCACGCATTCCTTTTCTGT	79	59	NM_001032355.1
*Igf2*	GGCATCGTGGAAGAGTGCT	128	60	X56094.1
*Igf2*	CTGGGGAAGTTGTCCGGAAG	128	60	X56094.1
*Cdx2*	CAGCCAAGTGAAAACCAGGAC	119	59	NM_001278769.1
*Cdx2*	CGGCCTTTCTCCGAATGGT	119	60	NM_001278769.1
*ACTB*	AGATCGTGCGGGACATCAAG	93	59	DQ452569.1
*ACTB*	GCGGCAGTGGCCATCTC	93	59	DQ452569.1

### Statistical analysis

*In vitro* embryo development, differences in blastocyst cell numbers and embryo diameter were compared by Fisher’s exact test analysis. TUNEL assay and dead cell index were compared using T-Student test. Differences were considered to be significant at p< 0.05 for both studies. We performed the real time PCR with *ACTB* as reference gene. The relative expression of each gene were calculated from the average Ct values of each triplicate using the 2 (-ΔCt) method. Differences in the level of gene expression on both groups were analyzed using a parametric T-Student test. Differences between groups were evaluated with a significance level of p< 0.05. The data obtained in this study were analyzed using the GraphPad Prism statistical program (GraphPad Software, San Diego, CA).

## Results

In the present study, we aimed to elucidate if embryo aggregation improves *in vitro* cloning efficiency in the porcine, through a comprehensive study of the quality of the embryo. In order to evaluate this, embryo developmental rates, embryo diameter and cell number, DNA fragmentation levels and the pattern expression of different genes were analyzed.

*In vitro* embryo development records are shown in Table 2. We observed that cleavage rates per ZFREs were significantly higher in 3x aggregated cloned embryos than in the control group 1x, 92.17% *vs* 73.05% respectively, p<0.0001. However, blastocyst rate per ZFRE did not differ between groups. On the other hand, blastocyst rates per embryo (considering each well as an embryo) in the 3x group (37.39%) was more than three times that of the control group (11.38%) (p<0.0001). Zona free cloned embryo aggregation improved blastocyst rates, and did not involve the use of additional oocytes to obtain more embryos.

**Table 2:** Effects of clone porcine embryo aggregation on *in vitro* embryo development until day 7.

Experimental groups	No. ZFREs*	No. Embryos (wells)	No. cleaved ZFREs (%)	No. blastocyst	% of blastocyst per ZFREs	% of blastocyst per embryo (well)
**1x**	167	167	122 (73.05) ^a^	19	11.38 ^a^	11.38 ^a^
**3x**	345	115	318 (92.17) ^b^	43	12.46 ^a^	37.39 ^b^
**Total**	**512**	**282**	**440 (85.94)**	**62**	**12.11**	**21.99**

*ZFREs: Zona Free Reconstructed Embryos (a,b) Values from the same method with different superscripts in a column are significantly different (p<0.05, Fisher’s exact test).

Cloned blastocyst diameter was measured and embryos were divided in three different groups according to their diameter at day 7: 80 μm-199 μm (small), 200 μm-299 μm (medium) and > 300 μm (large). The numbers of embryos analyzed in each diameter category were: 1x (n = 16) and 3x (n = 28). Embryo diameters per group were: in the small 1x group n = 12: 99.6 μm (1), 199.2 μm (4), 132.8 μm (3), 166 μm (3) and 149.4 μm (1); in the medium 1x group n = 4: 1 of each value: 215.2 μm, 249 μm, 265.6 μm and 282.2 μm. In the small 3x group n = 7: 132.8 μm (3), 166 μm (3) and 199.2 μm (1); in the medium 3x group n = 12: 215.8 μm (2), 232.4 μm (2), 249 μm (4), 265.6 μm (2), 282.2 μm (1) and 298.8 μm (1). Finally, in the large 3x group n = 9: 365.2 μm (2), 381.8 μm (3), 415 μm (1), 448.2 μm (2) and 514.6 μm (1). Aggregated 3x cloned embryos were bigger than 1x non aggregated cloned embryos and these differences were statistically significant (p<0.011). Experimental group 1x had a higher proportion (75%) of small diameter embryos, and no blastocyst measured more than 300 μm. Conversely, 75% of cloned pig aggregated blastocysts (experimental group 3x) were of medium and large diameters. Results are shown in Table 3.

**Table 3:** Effects of embryo aggregation on *in vitro* porcine cloned embryo diameter.

Experimental groups	No. Blastocyst	Blastocyst diameter		
		80 μm- 199 μm (%)	200 μm- 299 μm (%)	>/ = 300 μm (%)
**1x**	16	12(75,0)^a^	4 (25,0)	0 (0) ^a^
**3x**	28	7 (25,0)^b^	12 (42,86)	9 (32,14)^b^
**Total**	**44**	**19 (43.18)**	**16 (36.36)**	**9 (20.45)**

**Blastocyst diameter of cloned aggregated and non-aggregated embryos at day 7.**Values with different superscripts in a column are significantly different (p<0.05, Fisher’s exact test).

**Fig 1:**
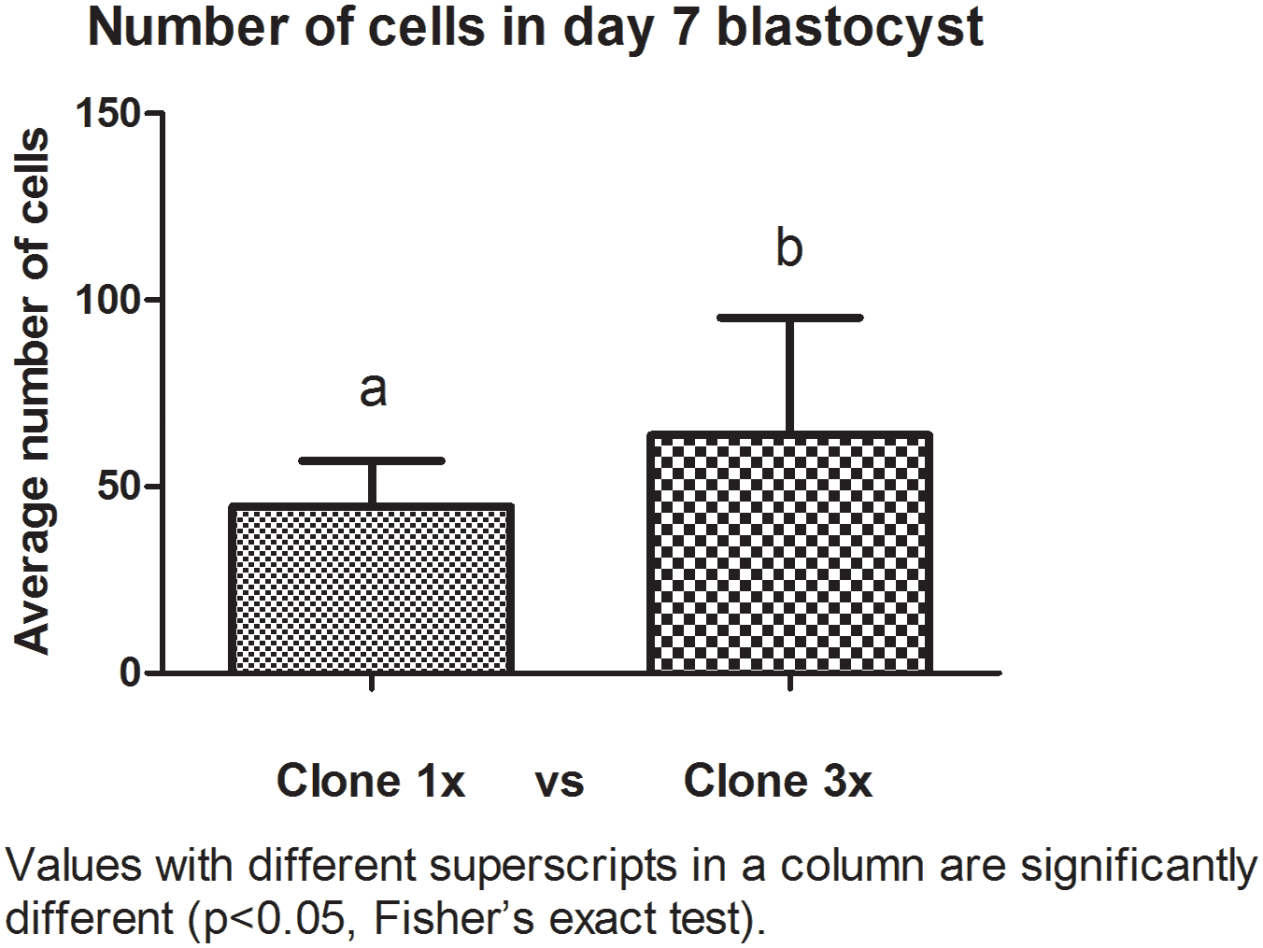
Blastocyst number of cells of cloned aggregated and non-aggregated embryos at day 7. Nuclei were counterstained with 30μg/mL propidium iodide (P4170) for 10 min in the dark.

Cell numbers of day 7 cloned blastocysts were different among groups. The aggregated embryos had more cells than the non aggregated embryos. The average number of cells in 1x group was between 27–64 cells and in the 3x group between 26–108 cells (Fig 1). More of the 3x blastocysts were larger (> 300μm) compared with the 1x blastocysts (p< 0.0001) (Table 3).

**Fig 2:**
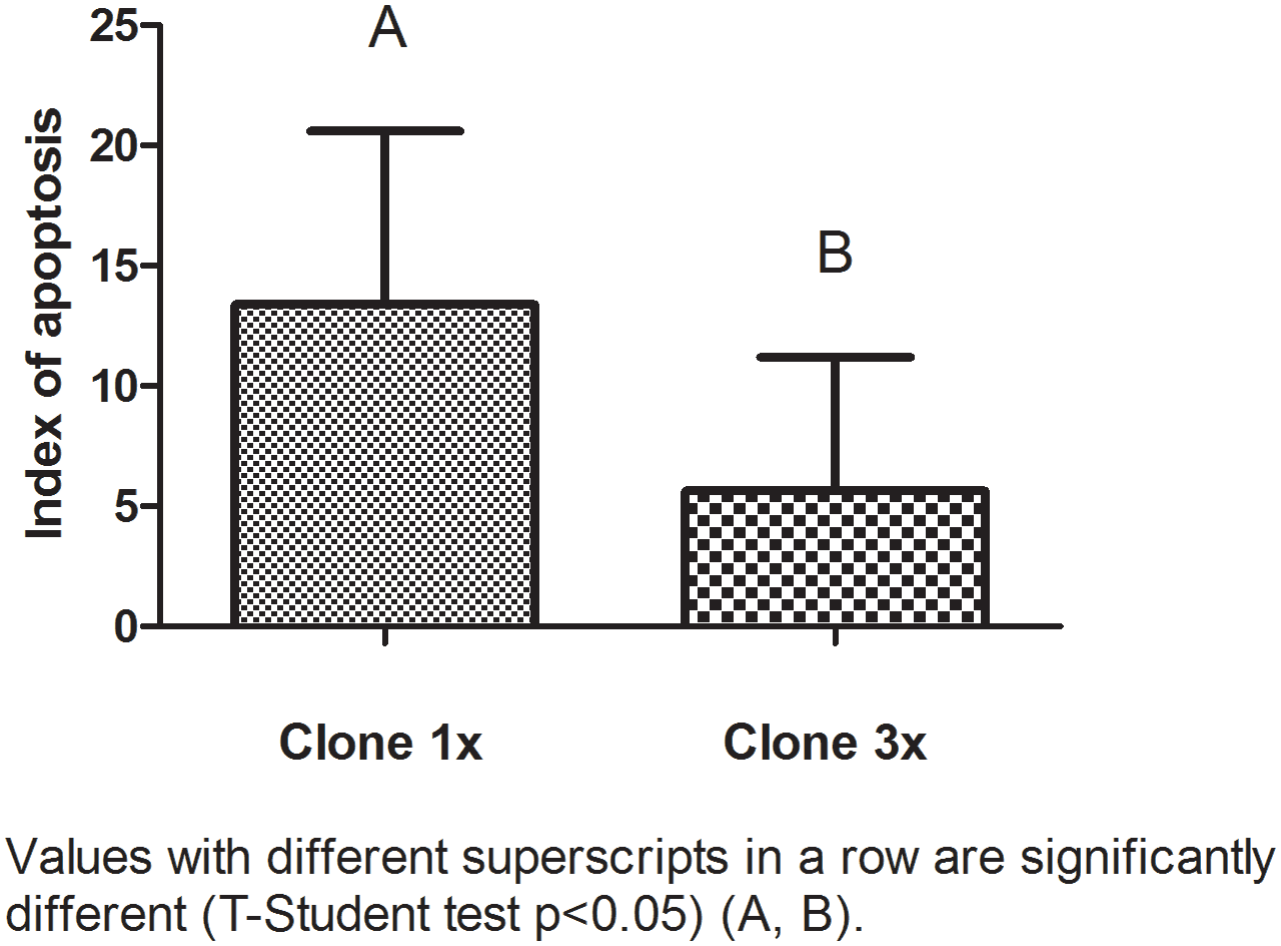
Index of apoptosis of aggregated and non-aggregated pig cloned blastocyst, showing statistically differences (p<0.05, T-Student test).

**Fig 3:**
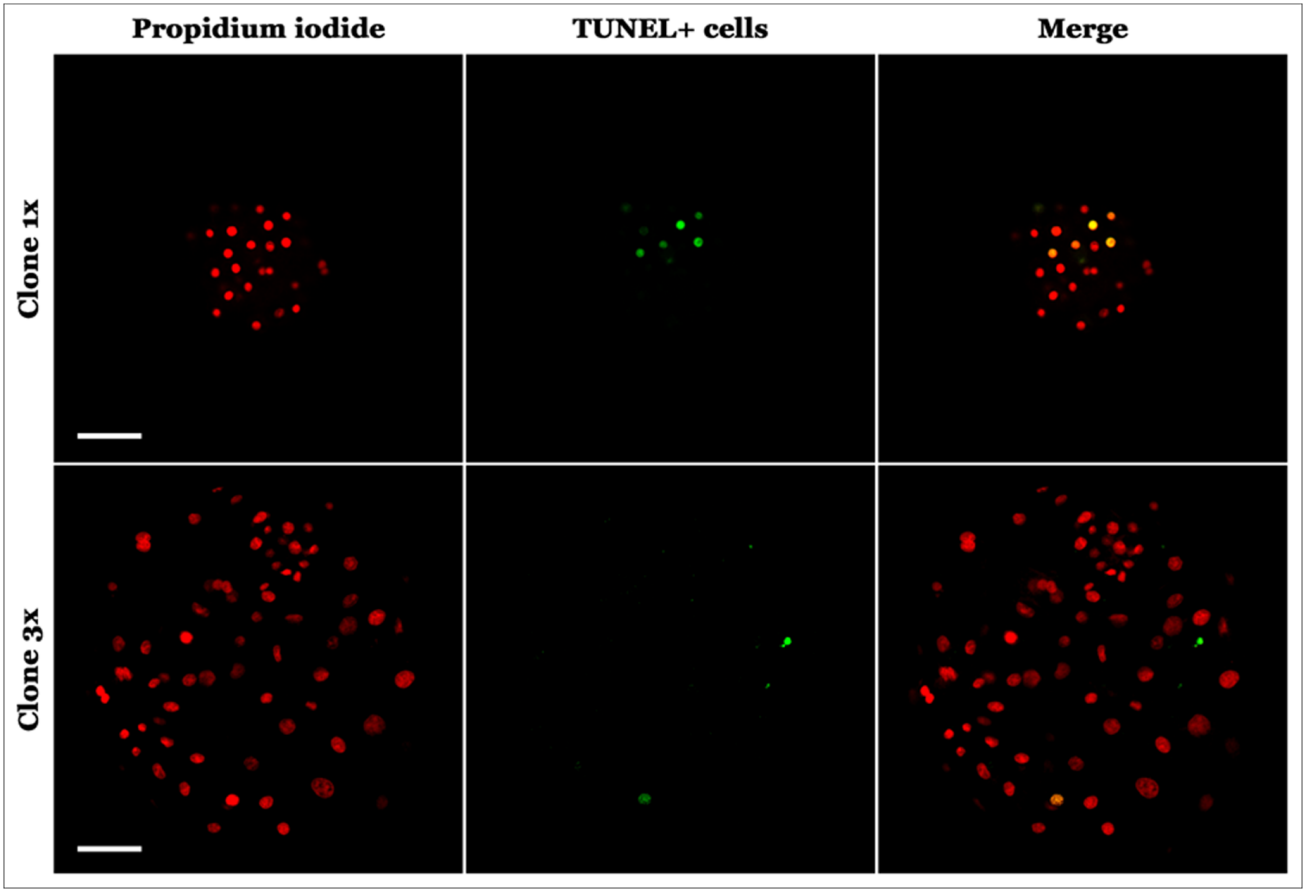
Photomicrographs of day 7porcine cloned embryo expression of TUNEL. Above non-aggregated cloned porcine embryo, 40x zoom. Below (day 7) 3x aggregated cloned embryo, 40x zoom.

The levels of apoptotic cells were determined by detection of fragmented DNA by TUNEL assay. The 3x group embryos had lower apoptotic index cells than the control group1x (4.18% vs 13.18%) (p<0.05). Results are shown in Figs 2 and 3 and Table 4.

**Table 4:** Index of apoptosis of aggregated and non-aggregated pig cloned blastocyst at day 7.

Cloned 1x Blastocyst			Cloned 3x Blastocyst		
Total cell number	Apoptotic cell number	IAP (Index of Apoptosis)	Total cell number	Apoptotic cell number	IAP (Index of Apoptosis)
27	4	14,81	26	2	7,69
42	4	9,52	24	4	16,67
54	4	7,41	66	3	4,54
54	5	9,26	38	2	5,26
64	9	14,06	68	8	11,76
27	4	14,81	89	1	1,12
49	12	24,49	108	3	2,78
43	10	23,25	103	1	0,97
42	1	2,38	52	0	0
**402**	**53**	**13,18**^**a**^	**574**	**24**	**4,18**^**b**^

Values with different superscripts in a row are significantly different (T-Student test p<0.05).

**Fig 4:**
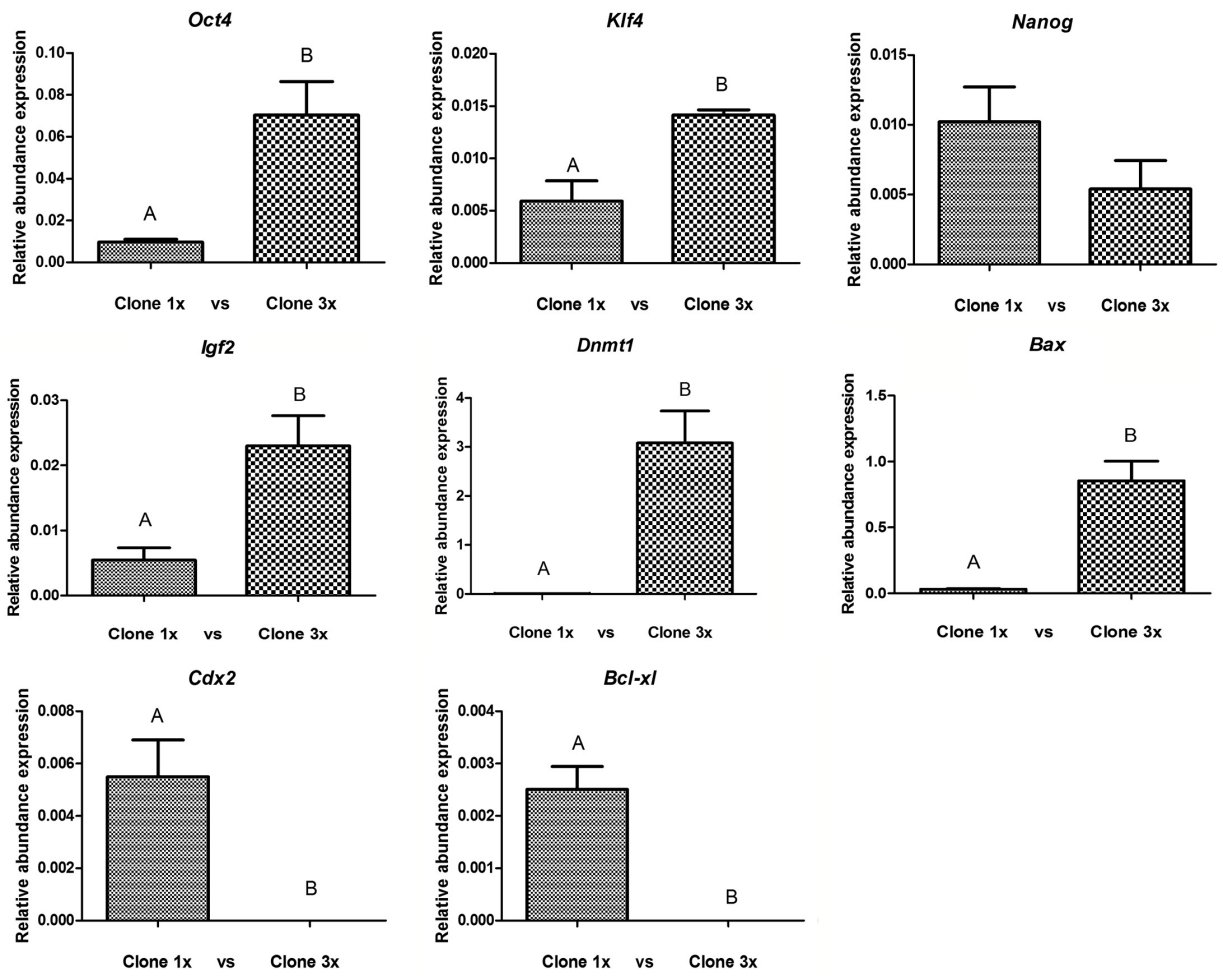
Relative transcript abundance of *Oct4*, *Klf4*, *Nanog*, *Igf2*, *Cdx2*, *Dnmt1*, *Bcl-xl*, *and Bax* genes in pig day 7 blastocysts generated by SCNT. All genes were normalized with the ***ACTB*** gene. (A, B) different letters are significantly different within each gene expression (p<0.05).

In order to evaluate the effect of cloned embryo aggregation on cellular reprogramming, we measured mRNA of pluripotency genes: *Oct4*, *Klf4* and *Nanog*; two differentiation related markers *Cdx2* and *Igf2*; two apoptosis markers *Bcl-xl* and *Bax* and finally *Dnmt1*, a key modulator of DNA methylation. Primers designed for this study are listed in materials and methods. We observed a significant statistical increase in the relative expression of *Oct4*, *Klf4*, *Igf2*, *Bax* and *Dnmt1* genes. On the other hand, *Nanog* expression was not affected by embryo aggregation, whereas the relative amount of mRNA of *Cdx2* and *Bcl-xl* genes were not detected in the 3x aggregate group in 40 cycles of the qPCR. Results of gene expression are shown in Fig 4.

## Discussion

In this study, we combined the assessment of developmental competence in terms of cleavage, blastocyst rate, number of cells, embryo diameter and DNA fragmentation levels to assess the effects of cloned pig embryo aggregation. Additionally, we performed a quantitative analysis of relative mRNA abundance of different genes involved in cell reprogramming (*Klf4*, *Nanog*, *Oct4*, *Bcl-xl*, *Bax*, *Igf2*, *Dnmt 1* and *Cdx2* compared with an endogenous gene: *ACTB*).

There are some limitations for cloning technique, such as the need of several good quality embryos to produce and maintain a pregnancy [1]. Furthermore, porcine oocytes and embryos are even more sensitive to temperature fluctuations than those from other domestic species [1]. It is believed that for a successful cloning, the pattern of epigenetic modifications in the donor cells must be remodeled to be similar to the pattern present in the fertilized embryos [35].As a result, we performed embryo aggregation and obtained a significant increase in *in vitro* embryo development and cell number, 20% cleavage and a three-fold increase in blastocyst rate. Similar observations were also reported following embryo aggregation in domestic animals such as the pig following aggregation of 2 cells [33], or of 4 cells [11, 14, 36, 37] and in the bovine [17], the horse [8, 9] and the feline [10]. Increased cleavage, development rates and cell number proved that the medium used was adequate for normal development. This is the first report where SOF medium was used for porcine embryos; it demonstrated a comparable rate of embryo development to other commonly used media.

We observed positive effects of embryo aggregation. This situation could be due to a) an epigenetic compensation since some epigenetic defects during cell reprogramming could be overcome by the combination of three embryos derived from the same cell line but with epigenetic differences; b) an increase in embryo cell number at day 0 of their development and a larger embryonic volume in the microwell system that may help embryos compact more easily, or c) a microenvironment generated by the interaction of beneficial and paracrine factors liberated by the aggregate embryo could increase rates of embryonic development; or d) a combination of all those situations. Additionally, embryo aggregation has been proposed as an alternative to avoid the increased level of heteroplasmy caused by the fusion of enucleated oocytes during the handmade cloning procedure [12, 16, 18]. In general, the term "heteroplasmy" refers to different mtDNA genomes in a single cell. Besides the minimal heteroplasmy produced by fusing a somatic cell into an enucleated oocyte during SCNT, embryo aggregation does not imply higher levels of heteroplasmy [38]. However, the potential effect of different mitochondrial DNA among cells but not within a cell generated by aggregated cloned embryos needs further investigation.

Based on results from the TUNEL assays, apoptotic cell indices were statistically lower in the 3x aggregated group compared with non-aggregated group. Lower levels of apoptotic cells in aggregated embryos were also reported for the pig [39] and the cow [40]. However, in other species, apoptotic levels do not change with embryo aggregation [9]. This situation may highlight that embryo aggregation effects depend on the embryo physiology characteristics of each species. Additional recent observations agree with our study, indicating that pig embryo aggregation has an anti-apoptotic effect due to fewer numbers of apoptotic cells as seen by TUNEL assay [14]. Embryo aggregation at day 0 increased embryo quality not only by increasing blastocyst diameters and initial number of cells during early embryo development but also by reducing the amounts of cells with fragmented DNA.

Unexpectedly, the relative expression of apoptotic related genes in the 3x experimental group is not necessary correlated with the observations obtained with the TUNEL assay. *Bcl-xl*, an anti apoptotic associated gene in cloned pig aggregated embryos was extremely low and could not be detected in 40 cycles of RT-qPCR and *Bax* (pro apoptotic associated gene) expression was significantly increased.

mRNA degradation is controlled by rates of synthesis and of decay [41]. However, recent results indicate that numerous untranslated mRNAs are assembled into “P bodies”, this consist in numerous messenger ribonucleoproteins that could accumulate in cytoplasmic foci and can be also degraded or return to translation [42– 45]. In eukaryotes, two general pathways of mRNA decay have been described and both pathways share the de-adenylation (removal of the polyA tail) [43, 46]. In aggregate embryos we saw increased numbers of cells and decreased protein translation showing less apoptosis in larger embryos. Mechanisms of this nature might be occurring in aggregated embryos. It remains unclear how this process is regulated in embryos, and how embryo aggregation could be involved. Further studies are required to understand the complete process.

As we mentioned previously, pluripotent related gene expression plays a critical role during cellular reprogramming and affects subsequent embryonic development [46]. Cloned embryos are reported to have lower expression of pluripotent genes when compared to *in vivo* embryos [47]. We observed higher expression of *Oct4*, *Klf4*, *Igf2* in aggregated blastocyst, suggesting that embryo aggregation at one cell stage could improve the pluripotent status at the blastocyst stage.

It has been reported in mice that aggregated clones have an improved expression of *Oct-4* and greater developmental potential compared with single clones [11]. Similarly, our results are in agreement with other reports in porcine aggregated blastocysts derived from SCNT embryos [33, 48]. Interestingly, embryo aggregation did not change *Nanog* expression while in previous studies expression levels of *Nanog* showed to be downregulated in cloned bovine blastocysts compared with their IVF counterparts [49, 50]. Surprisingly, we could not detect expression of *Cdx2* in aggregated embryos, possibly due to extremely low expression levels in this experimental group. Contradictory, it has been reported an increased expression of this gene in aggregated pig embryos [14]. The reported benefits of embryo aggregation in the establishment of pregnancies and subsequent *in vivo* embryo development [51], suggest that if *Cdx2* expression is affected in aggregated blastocyst, this may not affect future placentation. However, experiments focusing on placentation and *in vivo* embryo development of aggregated embryos are needed to determine the effects of embryo aggregation on blastocyst gene expression and its subsequent post implantation development.

Finally, *Dnmt1* (DNA cytosine-5 methyl-transferase) catalyzes the production and regulation of the dynamics of mammalian patterns of global genomic DNA methylation [52, 53, 54].Our results could involve redirection of methylase *Dnmt 1* in de novo methylation, involving reorganization of maternal methylation. The only way to verify if the rescheduling was successful, is to have pigs born alive, because the process of reprogramming the genome to turn into embryo is complicated and involves several steps, a process still not completely understood [54].

In conclusion, our data suggest that an incomplete reprogramming of porcine cloned embryos could be partially compensated by embryo aggregation, possibly by the combination of three different epigenetic embryos at the one cell stage supported by the higher expression of pluripotency genes observed in 3x aggregated embryos. Moreover, embryo aggregation improved the pre implantation *in vitro* developmental potential and increased blastocyst quality since diameter and cell number was higher while the level of apoptosis was lower.
